# A Suggested Equivalent Method for a Drainage Structure to Analyze Seepage in Tailings Dam

**DOI:** 10.3390/ma15207154

**Published:** 2022-10-14

**Authors:** Hongwei Zhang, Zhenzhong Shen, Detan Liu, Liqun Xu, Lei Gan, Yifei Long

**Affiliations:** 1 College of Water Conservancy and Hydropower Engineering, Hohai University, Nanjing 210098, China; 2 State Key Laboratory of Hydrology-Water Resources and Hydraulic Engineering, Hohai University, Nanjing 210098, China; 3 Datang Hydropower Science & Technology Research Institute Co., Ltd., Nanning 530007, China; 4 POWERCHINA Guiyang Engineering Croporation Limited, Guiyang 550081, China

**Keywords:** tailings dam, drainage structure, seepage analysis method, tailings discharge, phreatic line

## Abstract

To better understand the seepage field in tailings dam with a drainage structure that combines drainage mat, drainage tube, and geotextile, an equivalent seepage analysis method for the drainage structure is presented. In the method, an equivalent drainage structure is suggested to replace the original drainage. It has enough size to be easily presented in the three-dimensional (3d) model of a tailings dam. According to a back analysis procedure using the quasi-3d models of a tailings dam with original and equivalent drainage structures, the material properties of the equivalent drainage structure can be obtained under the principle of drainage capacity equivalence. It is demonstrated that the suggested method is accurate enough to capture the seepage field in a tailings dam based on comparing the calculated and measured phreatic lines in a tailings dam for verification. Then, the method is employed to investigate the seepage field in a tailings dam in China for a case study. The rise of water level, damage of drainage structure, or increase of tailings discharge speed and time will lift up phreatic line. After terminating tailings discharge, phreatic line will first rise and then fall. The effect of tailings discharge on phreatic line will almost disappear after terminating tailings discharge for 24 h.

## 1. Introduction

A tailings dam, a large-scale and manufactured structure, is built to store mine tailings and industrial waste for isolating tailings and preventing pollution of the surrounding environment. It also can leach slag and provide recycled water. Many tailings dams have been built around the world to match the production of the mining industry. According to the statistics, more than 24,605 tailings ponds were built on the global scale by 2021 [1], and the storage capacity of single tailings dam has reached 150.0 million $m^{3}$ [2]. There were more than 12,000 tailings ponds, including 4910 dangerous and disease ponds in China by 2018 [3]. By 2019, the United States had built more than 1400 tailings dams. It is necessary to underline that many small-scale tailings storage facilities have not been counted [4].

Because a tailings dam may cause major safety hazards [5], it is classified as one of the high-risk sources in China’s industry [6]. The tailings dam failure will cause serious loss of people’s life and property, and destroy the surrounding environment [7]. For example, the failure of the Pingdong tailings pond in China caused 277 deaths, 4 missing, and 33 injuries [8] in September 2008. In November 2015, the Fundão tailings dam in Brazil collapsed. The accident spilled about 32.6 million cubic meters of Germano iron mining. It killed 19 people and polluted 668 km of area involving the Atlantic Ocean and the Brazilian Atlantic Forest. It directly or indirectly affected the Sela & Tulipa and Selinha dikes, which are the main tailings dam in Brazil [9,10]. In March 2017, the event of a tailings dam failure in China killed 2 people and injured 6 people [11]. A tailing dam consisting of iron ore tailings in Brumadinho city, Brazil failed in January 2019, which caused the death of 259 and the missing of 11 by January 2020 [12,13]. The collapse of Brumadinho dam released tons of toxic sludge which polluted about 5.0 ${\mathrm{km}}^{2}$ area including the Paraopeba River [14].

Avoiding the failure of tailings dams has long been an area of focus for research [15,16]. After many summaries and investigations on tailings dam failures [16,17,18], seepage is considered one of the main causes of tailings dam failure [19,20]. Rodríguez et al. [15] investigated 67 tailings dam failures in Spain. Enhancing drainage measurement was considered to be instrumental in preventing tailings dam failure. The authors of Lyu et al. [21] listed the 40 key failures of tailings dams from 1928 to 2015 on the global scale, and the tailings dam failures directly induced by seepage accounted for 35% of total dam failures. Moreover, the seepage field has a direct and indirect effect on slope stability [22,23,24], internal erosion [13,19], and the pollution of environment including water and soil [17,25]. Hence, it is a critically important aspect that seepage control is implemented in the design, construction, and operation of a tailings dam [26].

**Figure 1:**
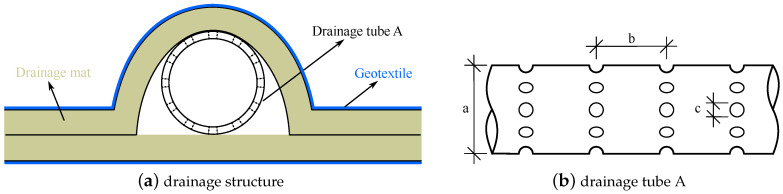
The drainage structure combining drainage mat, drainage tube, and geotextile, and the schematic diagram of drainage tube A.

In order to control seepage in a tailings dam, there exist some drainage measures in an actual tailings dam, such as drainage gravel, blanket drain [27], drainage mat [28], and drainage tube [29,30]. The distribution of these drainage structures is seriously complex. A drainage structure, combining drainage mat, drainage tube, and geotextile (see Figure 1a), is widely used in tailings dams in China, such as Baiyan tailings dam and Luchanggou tailings dam. The drainage tube with hole (see Figure 1b) is wrapped in the drainage mat, while the geotextile wraps the drainage mat. The thickness of the drainage mat is usually about 10.0~12.0 mm. The internal diameter *a* and hole diameter *c* of the drainage tube in Figure 1 usually are about 100.0 and 10.0 mm, respectively. Also, the geotextile is very thin. Hence, the thickness of the drainage structure is very small relative to the large-scale tailings dam.

Moreover, in terms of preventing the pollution of the surrounding environment, the landfill disposal of waste is consistent with the storage disposal of tailings. For draining leachate or lower groundwater, the drainage measures in waste disposal landfill sites are similar to that in a tailings dam [31]. The drainage tube in Figure 1b is also commonly used in waste disposal landfill sites [32].

**Figure 2:**
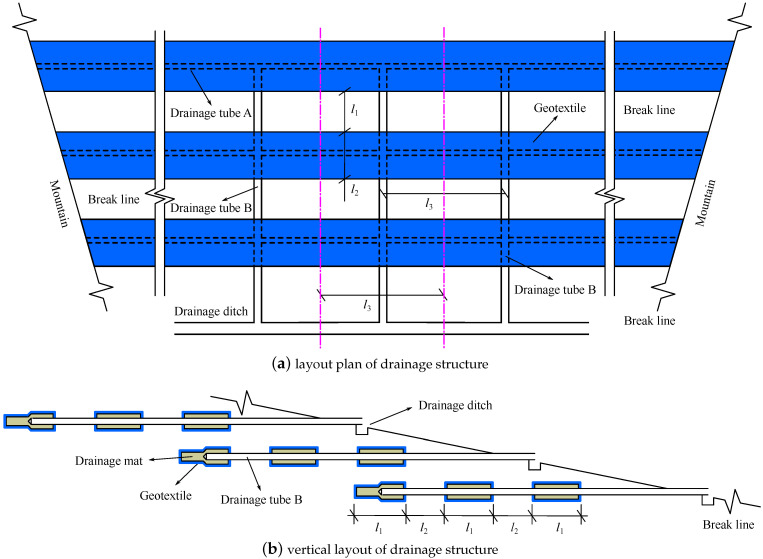
The layout of the drainage structure in a tailings dam. The drainage mat, geotextile, and drainage tube A are illustrated in Figure 1 in detail. The domain between two dash-dotted lines in Figure 2a is used to establish quasi-3d model.

The drainage structure in Figure 1 is usually laid in several rows at intervals of several meters in the vertical direction. Reference Figure 2 illustrates the layout of the drainage structure in tailings dam. There is no hole around drainage tube B. The drainage tube B is contacted with drainage tube A. The geotextile can effectively prevent tailings from the drainage mat. The water in the tailings first seeps into the drainage mat through the geotextile. Then it is collected into drainage tube A through the hole around drainage tube A. Finally, the water is drained from tailings pond through drainage tube B and transported to drainage ditch. This drainage structure usually constitutes a huge and complex drainage system in a tailings dam to ensure the water in the pond is drained well.

The seepage field in tailings dam should be evaluated during its service life. Numerical calculation is an important method to understand the seepage field in a tailings dam [27]. The drainage structure is usually simplified or ignored in an actual project considering convenient simulation, especially in 3d seepage model. For example, the Xiangyun tailings dam is simplified to be a homogeneous model after the neglect of drainage structure [33]. The drainage structure in the Lixi tailings dam is also not counted in the seepage analysis model [34]. The 3d model for seepage analysis of a tailings dam in [35] only consists of starter dam, tailings sand, and bedrock. Additionally, when the seepage analysis in waste disposal landfill sites is implemented, the drainage structure is also not considered [31]. However, although the size of drainage structure is much smaller than that of tailings dam, the drainage structure normally has good drainage performance. Thus, drainage structure should be considered in the numerical analysis model to accurately present the seepage field in a tailings dam.

The drainage structure in Figure 1 and Figure 2 is too small relative to the tailings dam, especially the drainage mat and geotextile. Due to the too small size and complex distribution of the drainage structure, it is challenging to generate the 3d numerical calculation model of tailings dam. In addition, the element size of the model will be too small if the drainage structure is analyzed in a tailings dam using conventional numerical method. Large numbers of elements will be required, leading to simulation efficiency being seriously reduced [36]. Equivalent medium method may be a good method to overcome the above difficulties for the seepage field in tailings dam with the drainage structure. However, there are no reported equivalent drainage structure types to replace the drainage structure combining drainage mat, drainage tube, and geotextile. Therefore, it is essential to present an equivalent drainage structure for the drainage structure in Figure 1 and Figure 2 to better evaluate the seepage field in a tailings dam.

In this paper, to better understand the seepage field in a tailings dam with the drainage structure in Figure 1, an equivalent seepage analysis method for the drainage structure is suggested. In the method, the original drainage structure is replaced by an equivalent drainage structure. To ensure the drainage capacity of the equivalent drainage structure equals the original drainage structure, a back-analysis procedure is used to obtain the material properties of the equivalent drainage structure. Then, the equivalent drainage structure can be employed to investigate the seepage field in the tailings dam. It is verified that the equivalent seepage analysis method for the drainage structure has a good accuracy through a tailings dam with in-situ measured phreatic lines. The equivalent seepage analysis method is easy and convenient to implement seepage analysis of a tailings dam with the drainage structure combining drainage mat, drainage tube, and geotextile.

## 2. Introduction of Saturated-Unsaturated Seepage Theory

### 2.1. Government Equations

Darcy’s law is accepted to describe the water flow in the unsaturated and saturated zone of tailings dam in this paper. When the compressibility of water and the influence of void gas on water flow movement are ignored, the heterogeneous anisotropic saturated–unsaturated seepage model can be expressed as [37,38]:

(1) $$\nabla \cdot \left(k_{r}\left(h_{c}\right)k\nabla \left(h_{c}+z\right)\right)-Q=\left(C\left(h_{c}\right)+\beta S_{s}\right)\frac{\partial h_{c}}{\partial t}$$

where ∇·(∗) and ∇(∗) denote divergence and gradient of (∗), respectively. k is the saturated hydraulic conductivity matrix. $h_{c}$ is the pressure water head, and *z* denotes the elevation head which is assumed to have nothing to do with time *t*. $k_{r}\left(h_{c}\right)$ is the relative hydraulic conductivity which ranges in (0, 1) in the unsaturated zone and $k_{r}\left(h_{c}\right)=1$ in the saturated zone. *Q* is the source term. $C\left(h_{c}\right)$ is the specific water capacity which will be zero in the positive pressure zone. β is a constant. In particular, β=0 and β=1 represent the unsaturated and saturated zone, respectively. $S_{s}$ is the elastic water storage rate. $k_{r}\left(h_{c}\right)$ and $C\left(h_{c}\right)$ can be expressed as follows according to van Genuchten [39].

(2) $$k_{r}\left(h_{c}\right)=\frac{{\left(1-{\left(\alpha h_{c}\right)}^{n-1}{\left(1+{\left(\alpha h_{c}\right)}^{n}\right)}^{-m}\right)}^{2}}{{\left(1+{\left(\alpha h_{c}\right)}^{n}\right)}^{m/2}}$$

(3) $$C\left(h_{c}\right)=\alpha \left(\theta_{r}-\theta_{s}\right)\left(n-1\right){\left(\alpha h_{c}\right)}^{n-1}{\left(1+{\left(\alpha h_{c}\right)}^{n}\right)}^{1/n-2}$$

where α and *m* are parameters that can be obtained from experimental results. *m* ranges (0, 1) and $n=\frac{1}{1-m}$. $\theta_{s}$ and $\theta_{r}$ are the saturated water content and the residual water content, respectively.

The governing Equation (1) is completed with the following a set of boundary conditions:

(4) $$\begin{array}{ccc} \; & h_{c}\left(x,t\right)=h_{c1}\left(x,t\right),\; & \mathrm{on}\;\Gamma_{1}\\ \; & -\left(k_{r}\left(h_{c}\right)k\nabla \left(h_{c}+z\right)\right)n=q_{n},\; & \mathrm{on}\;\Gamma_{2}\\ \; & -\left(k\nabla \left(h_{c}+z\right)\right)n\geq 0\;\mathrm{and}\;h_{c}\left(x,t\right)=0,\; & \mathrm{on}\;\Gamma_{3}\\ \; & -\left(k_{r}\left(h_{c}\right)k\nabla \left(h_{c}+z\right)\right)n=q_{r}\left(t\right)\;\mathrm{and}\;h_{c}\left(x,t\right)<0,\; & \mathrm{on}\;\Gamma_{4} \end{array}$$

in which $h_{c1}\left(x,t\right)$ is the known pressure head. n is the unit normal outward vector of boundary surface. $q_{n}$ and $q_{r}\left(t\right)$ are the normal flow and flow flux of infiltration, respectively. $\Gamma_{1}$, $\Gamma_{2}$, $\Gamma_{3}$, and $\Gamma_{4}$ denote the known water head boundary, known flow boundary, saturated overflow boundary, and unsaturated zone overflow boundary, respectively.

**Figure 3:**
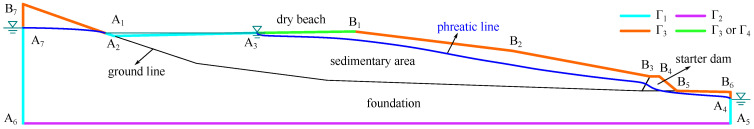
The boundary conditions of a conceptual tailings dam. $\Gamma_{1}$, $\Gamma_{2}$, $\Gamma_{3}$, and $\Gamma_{4}$ represent the known water head boundary, known flow boundary, saturated overflow boundary, and unsaturated zone overflow boundary, respectively.

A conceptual tailings dam (see Figure 3) is employed to illustrate the seepage boundary conditions in Equation (4) in this paper. Following the works [33,34,35,36], the line A${}_{1}$A${}_{2}$A${}_{3}$ represents the bottom of reservoir, and it will suffer from water head due to the catchment of reservoir. The lines A${}_{4}$A${}_{5}$ and A${}_{6}$A${}_{7}$ belong to the upstream and downstream underground water head, respectively. Hence, the lines A${}_{1}$A${}_{2}$A${}_{3}$, A${}_{4}$A${}_{5}$, and A${}_{6}$A${}_{7}$ are the known water head boundary $\Gamma_{1}$. The saturated overflow boundary $\Gamma_{3}$ is assigned to lines B${}_{1}$B${}_{2}$B${}_{3}$B${}_{4}$B${}_{5}$B${}_{6}$A${}_{4}$ and A${}_{1}$B${}_{7}$A${}_{7}$, and the boundary B${}_{1}$A${}_{3}$ also belongs to $\Gamma_{3}$ when tailings discharge is not considered. The boundary B${}_{1}$A${}_{3}$ will belong to $\Gamma_{3}$ under the consideration of tailings discharge. The line A${}_{5}$A${}_{6}$ is regarded as the known flow boundary $\Gamma_{2}$.In terms of the lateral boundaries of 3d model, if these boundaries are lower than the groundwater level, they belong to $\Gamma_{1}$; otherwise, they belong to $\Gamma_{3}$.

In addition, the governing Equation (1) is supplemented with an initial condition as:

(5) $$h_{c}\left(x,0\right)=h_{c}\left(x,t_{0}\right)\;\mathrm{in}\;\Omega$$

where $t_{0}$ is the initial time.

### 2.2. Computation Implementation

The governing Equation (1), together with Equations (4) and (5), yields an initial/ boundary-value problem for pressure water head $h_{c}\left(x,t\right)$, which is discretized into Equation (6) using Galerkin’s method in calculation domain Ω and backward Euler method in time.

(6) $$\left(C+\frac{1}{\Delta t}S\right)h_{t+\Delta t}=F+\frac{1}{\Delta t}Sh_{t}$$

where

(7) $$\begin{array}{cc} \; & C=\int_{\Omega }B^{T}k_{r}\left(h_{c}\right)\mathrm{kB}d\Omega \\ \; & S=\int_{\Omega }N^{T}\left(C\left(h_{c}\right)+\beta S_{s}\right)Nd\Omega \\ \; & F=-\int_{\Omega }B^{T}k_{r}\left(h_{c}\right)k_{z}d\Omega -\int_{\Omega }N^{T}Qd\Omega -\int_{\Gamma_{2}}N^{T}q_{n}d\Gamma_{2}-\int_{\Gamma_{4}}N^{T}q_{r}d\Gamma_{4} \end{array}$$

in which Δt is time step size. h is the pressure water head on nodes. $h=Nh_{c}$. N is the shape function for pressure water head $h_{c}$. B is the derivative of the corresponding shape functions in N. The superscript *T* indicates matrix transpose.

## 3. Equivalent Seepage Analysis Method for Drainage Structure

### 3.1. Equivalent Seepage Analysis Method

According to the introduction in Section 1, there are two difficulties in investigating seepage field in tailings dam with the drainage structure in Figure 1 and Figure 2 using 3d numerical calculation. First, because the drainage structure is very complex and thin relative to the tailings dam, it is not easy to generate the 3d model of tailings dam. Second, the total element number related to calculation accuracy is limited under the consideration of calculation cost. Thus, a seepage analysis method for the drainage structure composed of geotextile, drainage mat, and drainage tube urgently needs to be proposed to better understand the seepage field in the tailings dam.

To overcome these two difficulties, an equivalent seepage analysis method for the drainage structure is expected, in which an equivalent drainage structure is used to replace the original drainage structure. The seepage analysis of the equivalent drainage structure should be easily implemented in the 3d model of the tailings dam, so the equivalent drainage structure must be simple in structure and thick enough. Moreover, it is crucially important that the drainage capacity of the equivalent drainage structure should be the same as that of the original drainage structure. This requirement can be satisfied by adjusting the material parameters of equivalent drainage structure based on a back-analysis procedure using quasi-3d models of tailings dam with original and equivalent drainage structures. If the water potential distributions in these two quasi-3d models match well, the equivalent drainage structure with corresponding material parameters has the drainage capacity of the original drainage structure.

**Figure 4:**
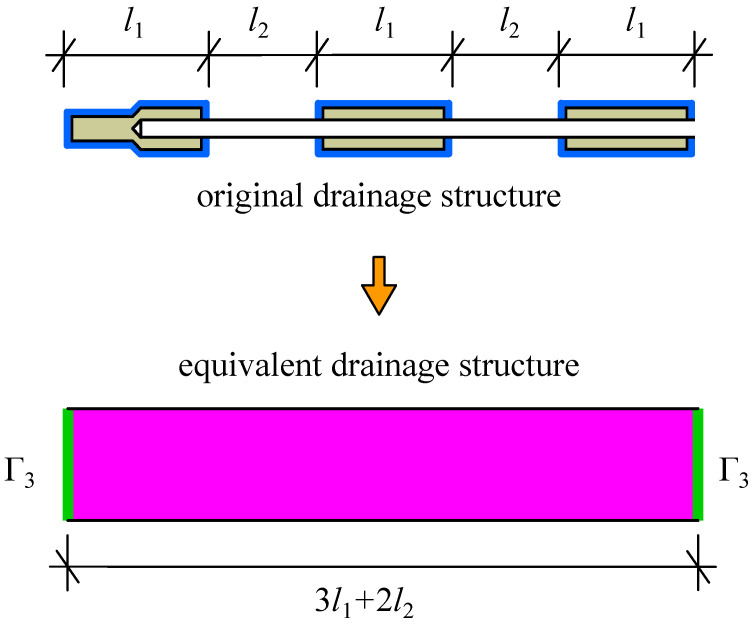
The suggested equivalent seepage analysis method for the drainage structure in Figure 2. $\Gamma_{3}$ is the saturated overflow boundary, expressed in Equation (4).

Therefore, to complete the seepage analysis of a tailings dam, the size of the equivalent drainage structure should be first determined, and then the material parameters of the equivalent drainage structure can be obtained based on back analysis. In this paper, it is suggested that the several rows of original drainage structures on the same layer are regarded as an entire equivalent drainage structure. For example, the 3 rows of original drainage structure on the same layer in Figure 2b can be replaced by an equivalent drainage structure with the width of $3l_{1}+2l_{2}$ (see Figure 4). The thickness of the equivalent drainage structure can be enlarged according to the size of the tailings dam. Also, the length of the equivalent drainage structure should be the same as that of the original drainage structure. When the quasi-3d model of a tailings dam with the equivalent drainage structure is implemented, the two lateral boundaries of equivalent drainage structure are regarded as the saturated overflow boundary condition $\Gamma_{3}$ (see Figure 4).

The suggested equivalent drainage structure has the function of the original drainage structure, i.e., water collection and drainage. The structure and boundary conditions of the equivalent drainage structure are simple, so it is easily implemented in the 3d model of tailings dam. After the size and properties of the equivalent drainage structure are determined, the equivalent drainage structure can replace the original drainage structure to participate in the 3d numerical calculation of tailings dam.

### 3.2. Verification of the Suggested Method

In this section, the suggested equivalent seepage analysis method for the drainage structure in Figure 2 will be verified via an actual tailings dam. The tailings dam has worked some years well. And it has in-situ measured phreatic lines. The permeability of the equivalent drainage structure is obtained through a back-analysis procedure by comparing the predicted seepage field in quasi-3d models of the tailings dam with the predicted seepage field using the original structure. Then, the permeability of the equivalent drainage structure is employed to calculate the phreatic line of the 3d model of the tailings dam with the equivalent drainage structure, and the calculated phreatic line will be compared with the measured phreatic line.

The accumulation level of the tailings dam for verification is 1392.0 m, and the corresponding total dam height is 199.0 m. Its normal water level is 1387.24 m. There are 7 layers of drainage mat, and every layer of drainage mat has three rows of drainage structure. The layout of the original drainage structure of the tailings dam for verification is illustrated in Figure 2 in which the $l_{1}=10.0$ m, $l_{2}=10.0$ m, and $l_{3}=50.0$ m. The thickness and width of the drainage mat are 10.0 mm and 10.0 m, respectively. The size of drainage tube A is as follows: a=100.0 mm, b=100.0 mm, and c=10.0 mm in Figure 1b. The internal diameter of drainage tube B is 150.0 mm.

**Figure 5:**
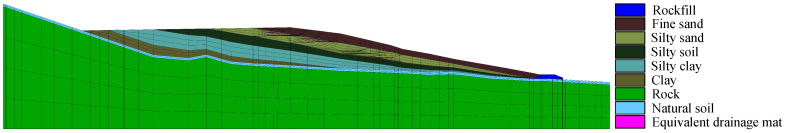
The typical section mesh of the quasi-3d model of tailings dam with the equivalent drainage structure to verify the suggested equivalent seepage analysis method.

The tailings in the pond can be partitioned into fine sand tailings, silty sand tailings, silty soil tailings, silty clay tailings, and clay tailings (see Figure 5). The 3 rows of the original drainage structure in the same layer are regarded as an equivalent drainage structure with a thickness of 2.0 m and a width of 50.0 m. According to the domain between two dash-dotted lines in Figure 2a, the quasi-3d models of the tailings dam with original and equivalent drainage structures are established. The typical section mesh of the quasi-3d model of tailings dam with the equivalent drainage structure is shown in Figure 5. To present the small original drainage structure, the mesh of the tailings dam with the original drainage structure is much finer than that of the tailings dam with the equivalent drainage structure. The mesh size of the tailings dam with the original drainage structure is too small, so the mesh is not presented in this paper. Note that the water in drainage tube A in Figure 2 can flow out freely through drainage tube B, so drainage tube A is modeled as the saturated overflow boundary $\Gamma_{3}$ (see Equation (4)). The drainage tube B and geotextile are not counted in the quasi-3d model of the tailings dam with the original drainage structure in this study. The boundary conditions in quasi-3d models are given based on the introduction in Section 2.1.

The material proprieties of the tailings dam for verification are listed in Table 1, and the permeability of materials is regarded as isotropic. The material permeability is dominant for the seepage field in a dam, so the object of back analysis is often just the permeability of material, even in saturated-unsaturated seepage analysis [37,38,40]. Hence, only the permeability of the equivalent drainage structure is inverted in this study. Its other properties are the same as that of the drainage mat. The equivalent drainage structure permeability is searched for in the range of $1.0\times 10^{-5}$∼$1.0\times 10^{-2}$ m/s.

**Figure 6:**
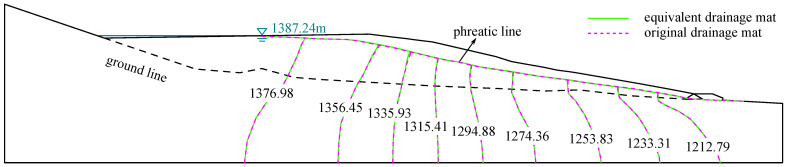
The water potential distributions in quasi-3d models for verification (m). The water potential distributions in the tailings dam with equivalent and original drainage structures are approximately identical when the permeability of equivalent drainage structure is $1.0\times 10^{-4}$ m/s.

Figure 6 presents the water potential distributions in the quasi-3d models with the equivalent and original drainage structures under the normal water level of 1387.24 m, and the equivalent drainage structure is $1.0\times 10^{-4}$ m/s. The water potential distributions in the tailings dam with equivalent and original drainage structures are approximately identical. This suggests that the equivalent drainage structure with the permeability of $1.0\times 10^{-4}$ m/s has the drainage capacity of the original drainage structure. Hence, the equivalent drainage structure with the permeability of $1.0\times 10^{-4}$ m/s can replace the original drainage structure to verify the accuracy of the suggested equivalent seepage analysis method for drainage structure.

Additionally, there are 5520 and 26,435 elements in the quasi-3d models with the equivalent and original drainage structures, respectively. The quasi-3d models with the equivalent and original drainage structures are calculated on a computer configured with Intel(R) Core(TM) i5-10400 CPU @ 2.90 GHz in a single-threaded environment. Their computational time is about 5 min and 35 min, respectively. This indicates that the suggested equivalent seepage analysis method can effectively reduce computational costs.

**Figure 7:**
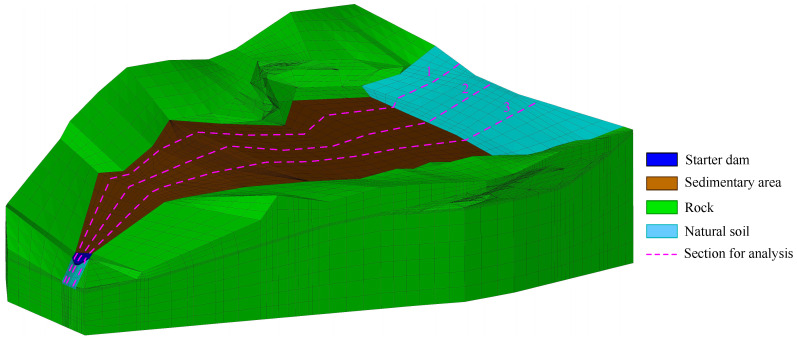
The 3d finite element mesh of the tailings dam with the equivalent drainage structure. The dashed lines are employed to illustrate the section location for analysis.

The 3d finite element mesh of the tailings dam for verification is shown in Figure 7. The three dashed lines are used to illustrate the section location for analyzing the seepage field. The tailings distribution in the sedimentary area can be checked in Figure 5. Table 1 shows the material properties. The permeability of equivalent drainage material is regarded as $1.0\times 10^{-4}$ m/s. The boundary conditions in the 3d model are similar to that in the quasi-3d model.

**Figure 8:**
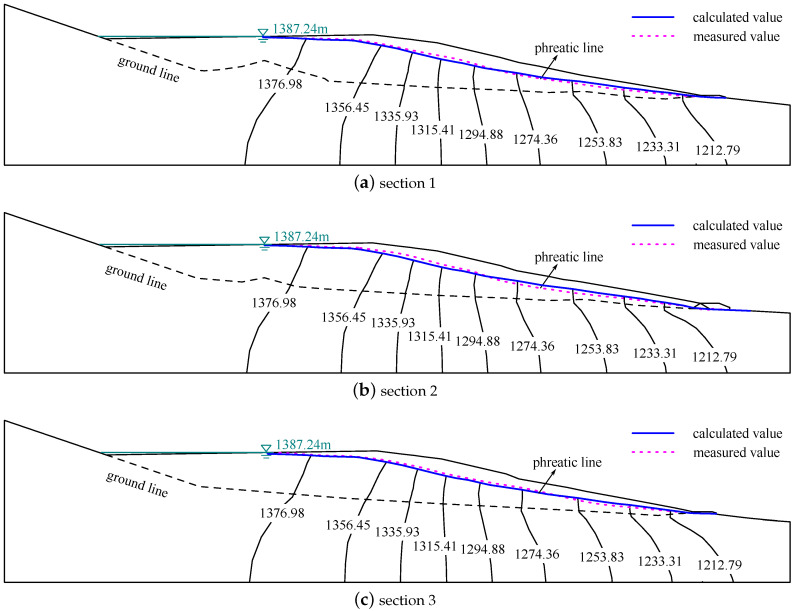
The measured and calculated phreatic lines of different sections (m). These sections are marked in Figure 7. The calculated phreatic line coincides with the measured phreatic line.

The measured and calculated phreatic lines of the different sections of the tailing dam are presented in Figure 8. It is observed that the calculated phreatic lines coincide with the measured phreatic line well. The maximum ratio of the difference between the measured and calculated value to the maximum water head is 3.85%, which is less than 5.0%. This suggests that the equivalent drainage structure with the permeability of $1.0\times 10^{-4}$ m/s can excellently capture the phreatic line in the tailing dam.

Therefore, the equivalent seepage analysis method for the drainage structure that is proposed can be used to investigate the seepage field in tailings dam. It is also shown that the permeability of the equivalent drainage structure can be predicted by comparing the predicted seepage results in quasi-3d models of a tailings dam with the predicted seepage results using the original drainage structure.

## 4. Case Study: Seepage Analysis of a Tailings Dam

The seepage field in a tailings dam is investigated in this section using the suggested equivalent seepage analysis method. Firstly, the basic information of the tailings dam is introduced. Secondly, the permeability of equivalent drainage structure is obtained according to back analysis. Thirdly, the effect of different design water levels, the damage of the drainage structure, and tailings discharge parameters on seepage field is analyzed.

### 4.1. Introduction of the Tailings Dam

**Figure 9:**
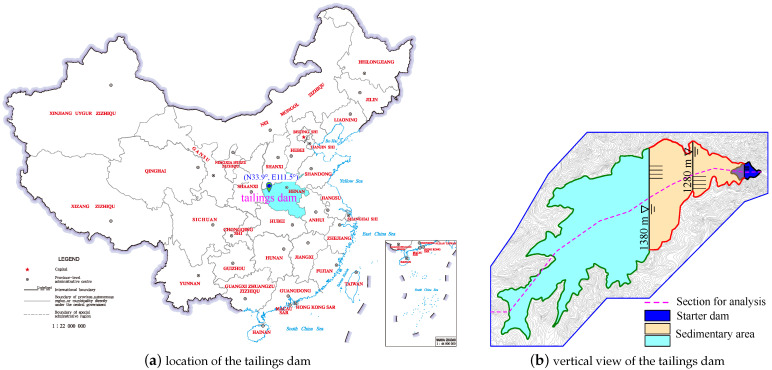
The location and vertical view of the tailings dam for a case study.

The tailings dam for a case study is located in Henan province in China (see Figure 9a). It is built in a V-shape valley. Its latitude and longitude are 33.9° N and 111.5° E, respectively. The region belongs to a warm continental monsoon climate, with an average annual precipitation of about 750.0 mm. The level of the starter dam is 1160.0 m, and its height is 79.0 m. The initial and effective storage capacity of the tailings dam are $401.0\times 10^{4}$$m^{3}$ and $293.0\times 10^{4}$$m^{3}$, respectively. The upstream embankment method is employed to build tailings embankment every 10.0 m along the elevation. The design accumulation level is 1380.0 m. The total tailings dam height and crest width are 299.0 m and 60.0 m, respectively. When the accumulation level reaches 1280.0 m, the crest width of the tailings dam is 30.0 m. The maximum distance between the submerged line of the tailings pond and the starter dam is about 2.9 km. The maximum tailings discharge speed is $4.0\times 10^{4}$ t/d. Figure 9b shows the vertical view of the tailings pond. Herein, because the tailings dam for the case study is located in China, the GB 50863-2013 [41], a code for designing tailings facilities in China, is used to determine the minimum buried depth of the phreatic line in this tailings dam. The minimum buried depth of the phreatic line must be more than 10.0 m.

There are 3 rows of original drainage structure very 15.0 m in the horizontal direction. The thickness and width of the drainage mat are 12 mm and 10.0 m, respectively. The *a*, *b*, and *c* in Figure 1b are 100.0, 100.0, and 10.0 mm, respectively. The drainage structure is laid on the inside of the mountain on both sides. The drainage tube B, with an internal diameter of 150.0 mm, is set very 50.0 m in the vertical direction along the dam axis. The drainage tube B connects with drainage tube A. The corresponding $l_{1}$, $l_{2}$, and $l_{3}$ in Figure 2 for the tailings dam are 15.0, 10.0, and 50.0 m, respectively. It may be noted that the drainage structures of a tailings dam for verification and case study are the same, but the sizes of their layout are different. Additionally, the in-situ measured phreatic lines in the tailings dam for the case study are unknown.

### 4.2. Permeability of Equivalent Drainage Structure

The suggested equivalent method for the drainage structure in Section 3 is accepted to analyze the seepage field in the tailings dam. The permeability of the equivalent drainage structure in this tailings dam is calibrated using back analysis method in this section.

**Figure 10:**
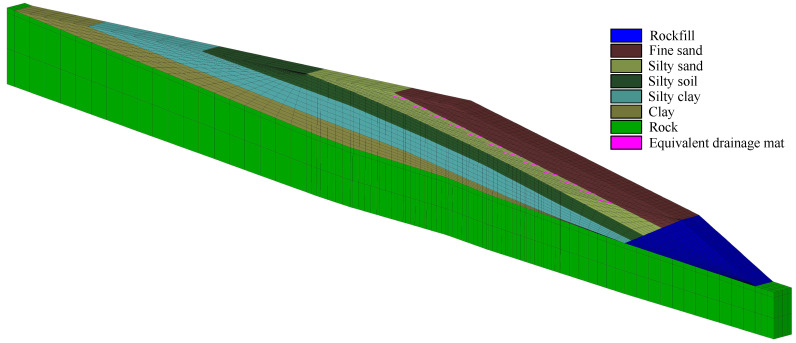
The quasi-3d finite element mesh of the tailings dam with equivalent drainage structure and the tailings distribution in the pond.

There are clay tailings, silty clay tailings, silty soil tailings, silty sand tailings, and fine sand tailings from the bottom to the top of sedimentary area. The tailings distribution is shown in Figure 10. The drainage structure is laid in silty sand tailings. The 3 rows of original drainage structure at the same layer in Figure 2b are regarded as an entirety to form the equivalent drainage structure with a width of 60.0 m and thickness of 1.0 m. The equivalent drainage structure is illustrated in Figure 10. The tailings dam with the accumulation height of 120.0 m is selected to analyze the permeability of the equivalent drainage structure, and the corresponding tailings dam level is 1280.0 m. The quasi-3d finite element mesh of the tailings dam with equivalent drainage structure is shown in Figure 10. The quasi-3d finite element mesh of tailings dam with original drainage structure is not presented because its mesh size is too small. The width of the quasi-3d model is 50.0 m, which is determined by the distance between two dash-dotted lines in Figure 2a. The model method of the original drainage structure in the quasi-3d model is the same as that in Section 3.2. Moreover, the boundary conditions in the quasi-3d models are assigned to be the same as that in Figure 3.

Table 2 shows the material properties of different zones in the tailings dam. The permeability of the equivalent drainage structure is looked for in $1.0\times 10^{-5}$∼$1.0\times 10^{-2}$ m/s to obtain an acceptable permeability. The other properties of the equivalent drainage structure are the same as that of the original drainage structure.

**Figure 11:**
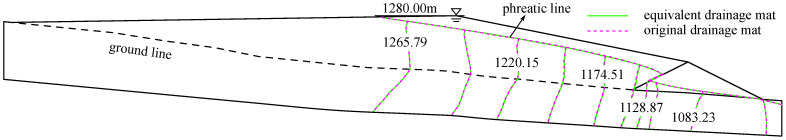
The water potential distributions (m) in the tailings dam with equivalent and original drainage structures when the permeability of equivalent drainage structure is $1.0\times 10^{-4}$ m/s. The plot suggests the water potential distributions in the tailings dam with the equivalent and original drainage structures are consistent.

Figure 11 shows the water potential distributions in the tailings dam with the equivalent and original drainage structures when the permeability of equivalent drainage structure is $1.0\times 10^{-4}$ m/s. It is observed that the water potential distributions in the tailings dam with the equivalent and original drainage structures are consistent, and it indicates that the drainage capacities of the original drainage structure and equivalent drainage structure with the permeability of $1.0\times 10^{-4}$ m/s are the same. Hence, the equivalent drainage structure with the permeability of $1.0\times 10^{-4}$ m/s can be used to capture the seepage behavior of the tailings dam.

The permeabilities of equivalent drainage structures for verification and case study are $1.0\times 10^{-4}$ m/s. The reason for this may be that the original and equivalent drainage structures of these tailings dams for verification and case study are different.

### 4.3. Seepage Analysis of the Tailings Dam

**Figure 12:**
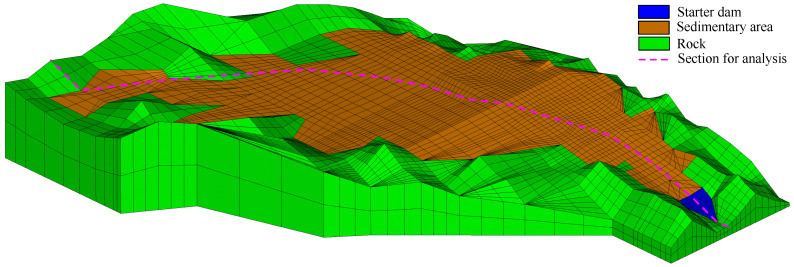
The 3d finite element mesh of the tailings dam with equivalent drainage structure. The equivalent drainage structure mesh of the section marked by the dashed line in this 3d mesh is the same as that in Figure 10. The tailings distribution in the sedimentary area is presented in Figure 10. The section is employed to analyze the seepage field in the tailings dam.

In order to investigate the seepage field in the tailings dam, the 3d mesh of the tailings dam is built, which is shown in Figure 12. Its boundary conditions are the same as that in the quasi-3d model in Section 4.2. The tailings distribution in the sedimentary area is the same as that in Figure 10. The material properties of different zones are listed in Table 2, and the permeability of equivalent drainage structure is $1.00\times 10^{-4}$ m/s, which is obtained in Section 4.2. Note that the section in the tailings dam used for analysis is illustrated in Figure 9b and Figure 12.

**Figure 13:**
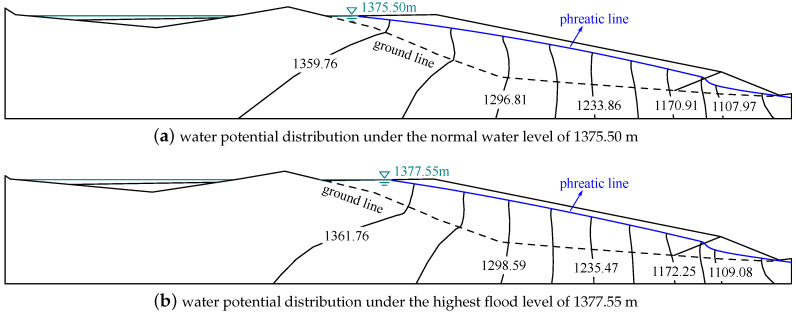
The water potential distributions in the section in the tailings dam under different water levels (m). The phreatic line under the highest flood level is higher than that under the normal water level, and the minimum buried depths of phreatic lines under these water levels address the requirement of the code [41].

Figure 13 shows the water potential distributions in the tailings dam under normal water level and highest flood level. The water in the pond flows out the tailings dam through the sedimentary area and starter dam.The phreatic line under the highest flood level is higher than that under the normal water level. The phreatic line minimum buried depth under normal water level and highest flood level is 25.41 and 23.09 m, respectively, and they satisfy the requirement of the code [41].

The hydraulic gradient in the tailings dam is not uniform. Generally, the hydraulic gradient in the middle of sedimentary area is larger than that in the zone near the top and bottom of the sedimentary area. The hydraulic gradient under normal water level and highest flood level is during 0.14~0.25 and 0.18~0.24, respectively.

**Figure 14:**
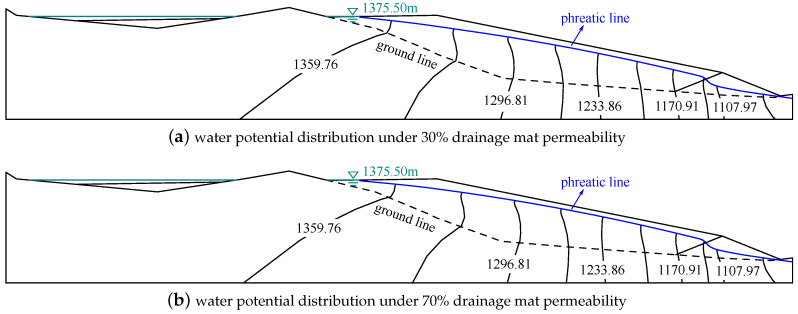
The water potential distributions under the different permeabilities of the equivalent drainage structure (m). The phreatic line will be lifted up if the drainage mat is damaged.

The original drainage structure may damage during the service life of the tailings dam. The damaged original drainage structure may not drain away the water well. In order to investigate the effect of drainage structure damage on the seepage field, the permeability of the equivalent drainage structure is decreased to 0.7 and 0.3 times of original value. Figure 14 presents the water potential distributions in the tailings dam with the different permeabilities of equivalent drainage structure under the normal water level of 1375.50 m. One can observe the phreatic line rises with the increase of the equivalent drainage structure permeability. This suggests that the phreatic line is lifted up when the drainage structure is damaged. A damage increase of the drainage mat leads to an increase in the phreatic line. Moreover, the phreatic line minimum buried depth of the tailings dam with 30% off and 70% off original permeability is 21.86 and 18.22 m, respectively. Those depths are more than the required phreatic line minimum buried depth for the safety design of tailings dam [41]. The maximum hydraulic gradient in the tailings dam with both 30% and 70% of original permeability is 0.27. The maximum hydraulic gradient in both cases is near the starter dam. This suggests that the drainage structure still has a good drainage performance when it has some damage in the investigated range.

Moreover, the effect of tailings discharge time and speed on the seepage field is investigated. Under the same tailings discharge condition, the lower the accumulation height of the tailings dam is, the greater the tailings discharge speed in the tailings pond is, and the greater effect of tailings discharge on the seepage field in the tailings dam is. Hence, the accumulation height is 20.0 m to analyze the seepage field in the tailings dam under different tailings discharge parameters, and the corresponding normal water level is 1175.50 m.

**Figure 15:**
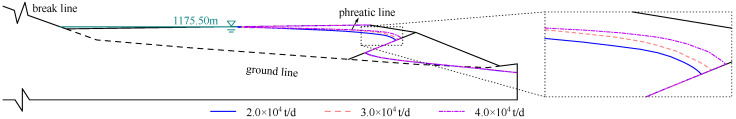
The effect of tailings discharge speed on phreatic line. With the increase of tailings discharge speed, the phreatic line rises.

Under the different tailings discharge speeds ($2.0\times 10^{4}$, $3.0\times 10^{4}$, and $4.0\times 10^{4}$ t/d), the seepage simulation of the tailings dam is implemented until seepage state reaches steady. The phreatic lines of the tailings dam in steady state are presented in Figure 15. The phreatic line is gradually lifted up with the increase of tailings discharge speed. The phreatic line minimum buried depth under the tailings discharge speed of $2.0\times 10^{4}$, $3.0\times 10^{4}$, and $4.0\times 10^{4}$ t/d is 19.72, 15.54, and 10.97 m, respectively.

**Figure 16:**
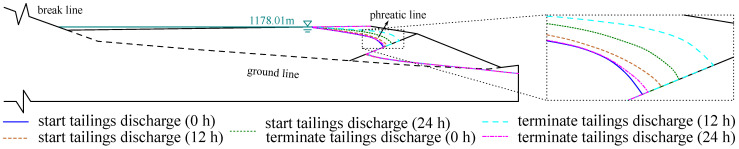
The effect of tailings discharge time on phreatic line. The phreatic line is lifted up with the increase of tailings discharge time. The phreatic line will first rise and then fall after terminating tailings discharge.

In order to investigate the effect of tailings discharge time on phreatic line, the tailings discharge is carried out for 24 h and then terminated. Figure 16 plots the phreatic lines of the tailings dam after starting tailings discharge for 0, 12, and 24 h and terminating tailings discharge for 12 and 24 h under the tailings discharge speed $3.0\times 10^{4}$ t/d and the highest flood level 1178.01 m. It illustrates that the phreatic line will be lifted up if the tailings discharge time increases. The minimum buried depth of phreatic line is 10.5 m after continuous tailings discharge for 24 h.

The phreatic line after terminating tailings discharge for 12 h is higher than that after terminating tailings discharge for 0 h. This indicates that the phreatic line continues to rise rather than fall immediately after the tailings discharge is terminated. The phreatic line after terminating tailings discharge for 24 h is close to the phreatic line after stating tailings discharge for 0 h, and it suggests that the effect of tailings discharge disappears approximately after terminating tailings discharge for 24 h. Hence, the phreatic line will first rise and then fall after terminating tailings discharge.

## 5. Conclusions and Recommendations

The drainage structure consisting of drainage mat, drainage tube, and geotextile is often used in tailings dam in China. It is challenging to obtain seepage field in tailings dam with the drainage structure. Hence, an equivalent seepage analysis method for the drainage structure is suggested to better investigate the seepage field in tailings dam.

In the suggested method, an equivalent drainage structure is employed to replace the original drainage structure. In order to ensure that the equivalent drainage structure has the drainage capacity of the original drainage structure, the permeability of the equivalent drainage structure is obtained based on back analysis using the quasi-3d models of tailings dam with original and equivalent drainage structures.

The in-situ measured phreatic line in a tailings dam is used to compare the calculated phreatic line using the suggested method. The calculated and measured phreatic lines are very consistent, which indicates that the suggested method can be adopted to study the seepage field in a tailings dam.

The suggested method is used to investigate the seepage field in a tailings dam in China. If the water level in pond rises, damage of the drainage structure occurs, or tailings discharge speed and time increase, phreatic line will be lifted up. The phreatic line will continue to rise during the initial stage after terminating tailings discharge. It will fall after tailings discharge is terminated for 12 h. After tailings discharge is terminated for 24 h, the effect of tailings discharge on phreatic line will disappear. When the permeability of the equivalent drainage structure reduces to 0.3 times of original value, the drainage structure still has a good drainage performance. In all investigated cases, the minimum buried depth of phreatic line is more than 10.0 m.

In this study, the parameters of equivalent drainage structure, except for permeability, are assumed to be the same as that of the original drainage structure. Although enough accurate seepage field can be captured under this assumption, all parameters of equivalent drainage structure should be obtained through a back-analysis procedure.

In conclusion, the suggested equivalent seepage analysis method for the drainage structure has enough accuracy to capture seepage field in tailings dam, and it is conveniently implemented in the 3d seepage analysis model.

## Figures and Tables

**Table 1:** The material properties of the tailings dam for verification.

Materials	Position	Permeability (m/s)	$\theta_{s}$ (%)	$\theta_{r}$ (%)	α (m${}^{-1}$)	*n*	$S_{s}$ (m${}^{-1}$)
Fine sand	Sedimentary area	$2.47\times 10^{-6}$	20	0.5	0.03	3	$1.0\times 10^{-9}$
Silty sand	Sedimentary area	$7.39\times 10^{-7}$	18	0.5	0.02	2.5	$1.0\times 10^{-9}$
Silty soil	Sedimentary area	$2.01\times 10^{-7}$	15	0.5	0.015	2	$1.0\times 10^{-9}$
Silty clay	Sedimentary area	$8.73\times 10^{-8}$	11	0.5	0.005	1	$1.0\times 10^{-9}$
Clay	Sedimentary area	$2.47\times 10^{-8}$	12	0.5	0.005	1	$1.0\times 10^{-9}$
Clay	Natural soil	$5.48\times 10^{-8}$	14	0.5	0.005	1	$1.0\times 10^{-9}$
Drainage mat	Sedimentary area	$1.00\times 10^{-3}$	50	0.1	0.5	10	$1.0\times 10^{-5}$
Rockfill	Starter dam	$3.00\times 10^{-6}$	10	2.0	0.5	10	$1.0\times 10^{-8}$
Rock	Dam foundation	$4.25\times 10^{-8}$	5	0.3	0.010	1.5	$1.0\times 10^{-9}$

**Table 2:** The material properties of different zones in the tailings dam for case study.

Materials	Position	Permeability (m/s)	$\theta_{s}$ (%)	$\theta_{r}$ (%)	α (m${}^{-1}$)	*n*	$S_{s}$ (m${}^{-1}$)
Fine sand	Sedimentary area	$2.47\times 10^{-6}$	20	0.5	0.03	3	$1.0\times 10^{-9}$
Silty sand	Sedimentary area	$7.39\times 10^{-7}$	18	0.5	0.02	2.5	$1.0\times 10^{-9}$
Silty soil	Sedimentary area	$2.01\times 10^{-7}$	15	0.5	0.015	2	$1.0\times 10^{-9}$
Silty clay	Sedimentary area	$8.73\times 10^{-8}$	11	0.5	0.005	1	$1.0\times 10^{-9}$
Clay	Sedimentary area	$2.47\times 10^{-8}$	12	0.5	0.005	1	$1.0\times 10^{-9}$
Drainage mat	Sedimentary area	$1.00\times 10^{-3}$	50	0.1	0.5	10	$1.0\times 10^{-5}$
Rockfill	Starter dam	$3.00\times 10^{-3}$	10	2.0	0.5	10	$1.0\times 10^{-8}$
Rock	Dam foundation	$4.60\times 10^{-11}$	5	0.3	0.012	1.2	$1.0\times 10^{-9}$

## Data Availability

Not applicable.
